# Supplementary material for: Sarcopenia assessed by 4-step EWGSOP2 in elderly hemodialysis patients: Feasibility and limitations
Source: PLoS One. 2022 Jan 13;17(1):e0261459. doi: 10.1371/journal.pone.0261459 (PMC8758069; doi:10.1371/journal.pone.0261459)
Supplement: S1 Table — (DOCX) [file pone.0261459.s001.docx]

| **Supplementary table 1. Demographic, anthropometric, analytical data and body composition by bioimpedance (mean ±SD).** | | | | |
| --- | --- | --- | --- | --- |
|  | **All**  **(n=60)** | **Male**  **(n=41. 68%)** | **Female**  **(n=19. 32%)** | ***p value** |
| ***Demographic data*** | | | | |
| Age (years) | 81.85±5.58 | 81.31 ±5.72 | 83±5.22 | 0.27 |
| Dialysis vintage (months) | 49.88±40.29 | 48.29±39.56 | 53.30±42.73 | 0.66 |
| ***Anthropometric data*** | | | | |
| Body mass index (kg/m^2^) | 25.20±3.64 | 25.73±3.40 | 24.08±3.95 | 0.10 |
| Mid-Upper Arm Circumference (cm) | 25.69±3.11 | 26.62±2.66 | 25.69±3.11 | 0.26 |
| Waist hip ratio | 0.92±0.08 | 0.95±0.69 | 0.85±0.06 | **<0.001** |
| ***Analytical data*** | | | | |
| Albumin (g/dl) | 3.66±0.47 | 3.69±0.41 | 3.59±0.60 | 0.43 |
| Hemoglobin (g/dl) | 11.26±1.13 | 11.40±1.02 | 11.26±1.13 | 0.63 |
| C Reactive Protein (mg/L) | 1.60±2.77 | 1.56±2.86 | 1.67±2.65 | 0.89 |
| 25OH Vitamin D_3_ (ng/ml) | 21.51±13.13 | 21.14±12.95 | 22.31±13.81 | 0.75 |
| Kt/V_urea_ | 1.80±0.38 | 1.70±0.372 | 2.01±0.30 | 0.001 |
| ***Body composition*** | | | | |
| Muscle Mass (kg) | 19.27±3.82 | 20.98±3.22 | 15.57±1.87 | **<0.001** |
| Fat Mass (kg) | 22.91±5.07 | 22.91±5.07 | 22±7.40 | 0.581 |
| Total Body Water (l) | 32.41±6.52 | 35.52±5.17 | 25.69±3.15 | **<0.001** |
| Overhydration (l) | 1. 10±1.41 | 1.26±1.61 | 0.75±1.21 | 0.233 |
| *p<0.05 in bold | | | | |
|  | | | | |
