# Supplementary material for: Sarcopenia assessed by 4-step EWGSOP2 in elderly hemodialysis patients: Feasibility and limitations
Source: PLoS One. 2022 Jan 13;17(1):e0261459. doi: 10.1371/journal.pone.0261459 (PMC8758069; doi:10.1371/journal.pone.0261459)
Supplement: S2 Table — (DOCX) [file pone.0261459.s002.docx]

| **Supplementary table 2. Sarcopenia criteria and prevalence of sarcopenia according to the individual components of 4-step EWGSOP2 steps in very elderly hemodialysis patients. Data presented as mean±SD or n (%).** | | | | | | | | |
| --- | --- | --- | --- | --- | --- | --- | --- | --- |
|  | **Individual criteria** | | | | **Prevalence of sarcopenia** | | | |
|  | **All**  **n=60** | **Male**  **n=41** | **Female n=19** | ***p**  **value** | **All**  **n=60** | **Male n=41** | **Female n=19** | ***p**  **value** |
| ***Find*** | | | | | | | | |
| **SARC-F** (points) | 2.6+ 2.3 | 2.5+ 2.4 | 2.8+ 2.3 | 0.56 | 18 (30%) | 12 (29%) | 6  (32%) | 0.86 |
| ***Assess*** | | | | | | | | |
| **GDS**  (Kg) | 19.2+6.6 | 21.68+5.9 | 13.92+4.5 | **<0.001** | 45 (75%) | 32 (78%) | 13 (68%) | 0.42 |
| **STS5**  (s) | 20.3+6.3 | 19.99+5.9 | 21.08+7.4 | 0.609 | 53 (88%) | 37 (90%) | 16 (84%) | 0.67 |
| ***Confirm*** | | | | | | | | |
| **ASM**  (kg) | 19.3+3.8 | 21.02+3.2 | 15.57+1.9 | **0.007** | 24 (40%) | 18 (44%) | 6  (62%) | 0.36 |
| ***Severity*** | | | | | | | | |
| **GS**  (m/s) | 0.69+0.27 | 0.70+0.26 | 0.67+0.30 | 0.67 | 42 (70%) | 30 (72%) | 12 (63%) | 0.43 |
| **TUG**  (s) | 19.1+12.1 | 19.47+13.2 | 18.31+9.5 | 0.75 | 22 (37%) | 15 (37%) | 7  (37%) | 0.98 |
| **SPPB** (points) | 6.2+2.9 | 6.38+2.9 | 5.88+2.9 | 0.55 | 45 (75%) | 32 (78%) | 13  (69%) | 0.42 |
| SARC-F: Strength, Assistance walking, Rise from a chair, Climb stairs, and Falls; GSD: grip strength by dynamometry, STS-5: sit to stand to sit 5, ASM: appendicular skeletal muscle mass, GS: gait speed, TUG: Timed-Up and Go test, SPPB: Short Physical Performance Battery. *p<0.05 in bold | | | | | | | | |
