# Supplementary material for: Sarcopenia assessed by 4-step EWGSOP2 in elderly hemodialysis patients: Feasibility and limitations
Source: PLoS One. 2022 Jan 13;17(1):e0261459. doi: 10.1371/journal.pone.0261459 (PMC8758069; doi:10.1371/journal.pone.0261459)
Supplement: S3 Table — (DOCX) [file pone.0261459.s003.docx]

| **Supplementary table 3. Correlation between the parameters of the EWGSOP2 evaluation and other variables in 60 very elderly hemodialysis patients.** | | | | | | | | |
| --- | --- | --- | --- | --- | --- | --- | --- | --- |
| **Variable** | | ***Find*** | ***Assess*** | | ***Confirm*** | ***Severity*** | | |
|  |  | **SARCF** (points) n=60 | **GSD**  (kg)  n=60 | **STS5**  (s)  n=43 | **ASM**  (kg)  n=60 | **GS**  (m/s) n=57 | **TUG**  (s)  n=53 | **SPPB**  (points) n=57 |
| **SARC-F**  (points) | *r  *p | --- | 0.454  **<0.001** | 0.210  0.176 | 0.313  **0.015** | 0.516  **<0.001** | 0.418  **0.001** | 0.423  **0.001** |
| **GSD**  (kg) | r  p | 0.454  **<0.001** | --- | -0.191  0.128 | 0.707  **<0.001** | 0.426  **<0.001** | 0.380  **0.005** | 0.490  **<0.001** |
| **STS5**  (s) | r  p | 0.210  0.176 | -0.191  0.128 | --- | -0.133  0.340 | -0.515  **<0.001** | 0.707  **<0.001** | -0.726  **<0.001** |
| **AMS**  (kg) | r  p | 0.313  **0.015** | 0.707  **<0.001** | -0.133  0.340 | --- | 0.159  0.104 | -0.187  0.179 | 0.217  0.104 |
| **GS**  (m/s) | r  p | 0.516  **<0.001** | 0.426  **<0.001** | -0.515  **<0.001** | 0.159  0.104 | --- | -0.884  **<0.001** | -0.798  **<0.001** |
| **TUG**  (s) | r  p | 0.418  **0.001** | 0.380  **0.005** | 0.707  **<0.001** | -0.187  0.179 | -0.884  **<0.001** | --- | -0.817  **<0.001** |
| **SPPB**  (points) | r  p | 0.423  **0.001** | 0.490  **<0.001** | -0.726  **<0.001** | 0.217  0.104 | -0.798  **<0.001** | -0.817  **<0.001** | --- |
|  | | | | | | | | |
| **Age**  (years) | r  p | 0.218  0.093 | -0.286  **0.026** | 0.259  0.093 | -0.396  **0.001** | -0.321  **0.015** | 0.354  **0.009** | -0.421  **0.001** |
| **Dialysis vintage**  (months) | r  p | 0.099  0.449 | -0.221  **0.089** | 0.151  0.334 | -0.064  0.625 | 0.002  0.986 | 0.090  0.521 | -0.145  0.281 |
| **Charlson**  (points) | r  p | 0.360  **0.004** | 0.016  0.902 | 0.348  0.223 | 0.159  0.225 | -0.380  **0.003** | 0.336  **0.013** | -0.332  **0.011** |
| **MIS**  (points) | r  p | 0.338  **0.008** | -0.264  **0.040** | 0.030  0.846 | -0.207  0.112 | -0.212  0.131 | 0.167  0.231 | -0.233  0.081 |
| **Barthel**  (points) | r  p | -0.726  **<0.001** | 0.483  **<0.001** | -0.378  **0.012** | 0.268  **0.037** | 0.529  **<0.001** | -0.418  **0.001** | 0.567  **<0.001** |
| **Frail**  (points) | r  p | 0.679  **<0.001** | -0.355  **0.005** | 0.291  0.059 | -0.179  0.170 | -0.477  **<0.001** | 0.329  **0.016** | -0.464  **<0.001** |
| **Body mass index** (kg/m^2^) | r  p | -0.076  0.56 | 0.537  **<0.001** | 0.144  0.357 | 0.474  **<0.001** | 0.084  0.532 | 0.099  0.478 | -0.141  0.292 |
| **Mid-Upper Arm Circumference** (cm) | r  p | 0.065  0.63 | 0.244  **0.007** | 0.132  0.40 | 0.400  **0.002** | 0.070  0.61 | -0.074  0.60 | -0.015  0.26 |
| **Waist hip ratio** | r  p | -0.242  0.071 | 0.377  **0.003** | -0.065  0.684 | 0.514  **<0.001** | 0.089  0.162 | -0.112  0.425 | 0.166  0.211 |
| SARC-F: Strength, Assistance walking, Rise from a chair, Climb stairs, and Falls; GSD: grip strength by dynamometry, STS-5: sit to stand to sit 5, ASM: appendicular skeletal muscle mass, GS: gait speed, TUG: Timed-Up and Go test, SPPB: Short Physical Performance Battery. Green hue marks functionality variables, while yellow hue marks anthropometry variables. Orange hue marks statistically significant p values for correlations between the parameters of the EWGSOP2 evaluation and functionality or anthropometry variables. *Within each cell, the r value is presented in the top row and the p value in the bottom row. *p<0.05 in bold. | | | | | | | | |
