# Supplementary material for: Sarcopenia assessed by 4-step EWGSOP2 in elderly hemodialysis patients: Feasibility and limitations
Source: PLoS One. 2022 Jan 13;17(1):e0261459. doi: 10.1371/journal.pone.0261459 (PMC8758069; doi:10.1371/journal.pone.0261459)
Supplement: Attachment — Submitted filename: Response to Reviewers.docx [file pone.0261459.s006.docx]

Additional Editor Comments:

The article has been improved and has the potential to be accepted. I agree with the discussion on sarcopenia/dynapenia. I agree with the Reviewer 1: all results according to gender have little added value (and are not discussed). At best, the results according to gender could be added in supplements. In the main text, I propose to focus on main results.

R: As suggested by the editor, we have now moved results by gender to suppl material. Additionally, the manuscript has been revised for English usage and typos.

Reviewers' comments:

Reviewer's Responses to Questions

**Comments to the Author**

1. If the authors have adequately addressed your comments raised in a previous round of review and you feel that this manuscript is now acceptable for publication, you may indicate that here to bypass the “Comments to the Author” section, enter your conflict of interest statement in the “Confidential to Editor” section, and submit your "Accept" recommendation.

Reviewer #1: (No Response)

Reviewer #2: All comments have been addressed

2. Is the manuscript technically sound, and do the data support the conclusions?

The manuscript must describe a technically sound piece of scientific research with data that supports the conclusions. Experiments must have been conducted rigorously, with appropriate controls, replication, and sample sizes. The conclusions must be drawn appropriately based on the data presented.

Reviewer #1: Yes

Reviewer #2: Yes

3. Has the statistical analysis been performed appropriately and rigorously?

Reviewer #1: Yes

Reviewer #2: Yes

4. Have the authors made all data underlying the findings in their manuscript fully available?

The [PLOS Data policy](https://eur02.safelinks.protection.outlook.com/?url=http%3A%2F%2Fwww.plosone.org%2Fstatic%2Fpolicies.action%23sharing&data=04%7C01%7CSMas%40fjd.es%7Cede8cca748f845d5791e08d9add69250%7Cd879fa4639544e5ca3cfab74f00c58da%7C0%7C0%7C637731959391709938%7CUnknown%7CTWFpbGZsb3d8eyJWIjoiMC4wLjAwMDAiLCJQIjoiV2luMzIiLCJBTiI6Ik1haWwiLCJXVCI6Mn0%3D%7C3000&sdata=g7VQifsQvs01qCjzTznb1DN540eHFitku%2FYmM2v7dHc%3D&reserved=0) requires authors to make all data underlying the findings described in their manuscript fully available without restriction, with rare exception (please refer to the Data Availability Statement in the manuscript PDF file). The data should be provided as part of the manuscript or its supporting information, or deposited to a public repository. For example, in addition to summary statistics, the data points behind means, medians and variance measures should be available. If there are restrictions on publicly sharing data—e.g. participant privacy or use of data from a third party—those must be specified.

Reviewer #1: Yes

Reviewer #2: Yes

5. Is the manuscript presented in an intelligible fashion and written in standard English?

PLOS ONE does not copyedit accepted manuscripts, so the language in submitted articles must be clear, correct, and unambiguous. Any typographical or grammatical errors should be corrected at revision, so please note any specific errors here.

Reviewer #1: No

Reviewer #2: Yes

6. Review Comments to the Author

Please use the space provided to explain your answers to the questions above. You may also include additional comments for the author, including concerns about dual publication, research ethics, or publication ethics. (Please upload your review as an attachment if it exceeds 20,000 characters)

Reviewer #1: 1. The reviewer still believe that there are too many statistical analyses in this manuscript and, for most of them, they do not refer at all to the main objective of this study (e.g. correlation between different sarcopenia categories and indexes).

R: We are in a difficult situation regarding this comment, since the table that referee is 1 referring to was requested by referee 2, who now thinks that the manuscript is appropriate for publication as is. We cannot satisfy the request by referee 1 without failing to satisfy the request from referee 2. As suggested by the editor, we have now moved results by gender to suppl material.

2. The authors refer now to dynapenia. Again, this is not the objective of the paper.

R: We agree that this was not the objective of the study. However, the concept of dynapenia was introduced to address comments by the referees and we believe that it helps to understand and discuss the results. Our results, in line with those of the literature, suggest a greater impact of uremia on dynapenia than on sarcopenia (at least when muscle mass is assessed by tools available for routine clinical care). Since some criteria used by EWGSOP2 do in fact assess dynapenia, we believe that the distinction between reduced muscle mass (i.e. sarcopenia) and decreased muscle strength (i.e, dynapenia) is important for the discussion of the results, even if this was not the aim.

Reviewer #2: The paper has significantly been improved with the provided modifications.
A typo error to be corrected: Pearson (and not Peasron) Page 8, line 187.

Corrected

7. PLOS authors have the option to publish the peer review history of their article ([what does this mean?](https://eur02.safelinks.protection.outlook.com/?url=https%3A%2F%2Fjournals.plos.org%2Fplosone%2Fs%2Feditorial-and-peer-review-process%23loc-peer-review-history&data=04%7C01%7CSMas%40fjd.es%7Cede8cca748f845d5791e08d9add69250%7Cd879fa4639544e5ca3cfab74f00c58da%7C0%7C0%7C637731959391709938%7CUnknown%7CTWFpbGZsb3d8eyJWIjoiMC4wLjAwMDAiLCJQIjoiV2luMzIiLCJBTiI6Ik1haWwiLCJXVCI6Mn0%3D%7C3000&sdata=hb6yz7kIufQ%2FoulCnVDRyZgZXRfjtllIsdeK37YO2CI%3D&reserved=0)). If published, this will include your full peer review and any attached files.


If you choose “no”, your identity will remain anonymous but your review may still be made public.


**Do you want your identity to be public for this peer review?** For information about this choice, including consent withdrawal, please see our [Privacy Policy](https://eur02.safelinks.protection.outlook.com/?url=https%3A%2F%2Fwww.plos.org%2Fprivacy-policy&data=04%7C01%7CSMas%40fjd.es%7Cede8cca748f845d5791e08d9add69250%7Cd879fa4639544e5ca3cfab74f00c58da%7C0%7C0%7C637731959391719926%7CUnknown%7CTWFpbGZsb3d8eyJWIjoiMC4wLjAwMDAiLCJQIjoiV2luMzIiLCJBTiI6Ik1haWwiLCJXVCI6Mn0%3D%7C3000&sdata=0ZsnEytrC5CDNHVHqamwyVnuU97V7twBfsfn2gBXAjg%3D&reserved=0).

Reviewer #1: No

Reviewer #2: **Yes:** Stanislas BATAILLE
